# Predictors of mortality among hospitalized COVID-19 patients and risk score formulation for prioritizing tertiary care—An experience from South India

**DOI:** 10.1371/journal.pone.0263471

**Published:** 2022-02-03

**Authors:** Narendran Gopalan, Sumathi Senthil, Narmadha Lakshmi Prabakar, Thirumaran Senguttuvan, Adhin Bhaskar, Muthukumaran Jagannathan, Ravi Sivaraman, Jayalakshmi Ramasamy, Ponnuraja Chinnaiyan, Vijayalakshmi Arumugam, Banumathy Getrude, Gautham Sakthivel, Vignes Anand Srinivasalu, Dhanalakshmi Rajendran, Arunjith Nadukkandiyil, Vaishnavi Ravi, Sadiqa Nasreen Hifzour Rahamane, Nirmal Athur Paramasivam, Tamizhselvan Manoharan, Maheshwari Theyagarajan, Vineet Kumar Chadha, Mohan Natrajan, Baskaran Dhanaraj, Manoj Vasant Murhekar, Shanthi Malar Ramalingam, Padmapriyadarsini Chandrasekaran

**Affiliations:** 1 Department of Clinical Research, ICMR-National Institute for Research in Tuberculosis (Formerly Tuberculosis Research Centre), Chennai, Tamil Nadu, India; 2 Department of General Medicine, Government Chengalpattu Medical College & Hospital, Chengalpattu, Tamil Nadu, India; 3 Department of Statistics, ICMR-National Institute for Research in Tuberculosis (Formerly Tuberculosis Research Centre), Chennai, Tamil Nadu, India; 4 Government Chengalpattu Medical College & Hospital, Chengalpattu, Tamil Nadu, India; 5 MDRU, Government Chengalpattu Medical College & Hospital, Chengalpattu, Tamil Nadu, India; 6 Department of Microbiology, Government Chengalpattu Medical College & Hospital, Chengalpattu, Tamil Nadu, India; 7 Department of Community Medicine, Government Chengalpattu Medical College & Hospital, Chengalpattu, Tamil Nadu, India; 8 Division of Epidemiology and Operational Research, ICMR-Vector Control Research Centre, Puducherry, India; 9 Central Leprosy Teaching & Research Institute, Chengalpattu, Tamil Nadu, India; 10 ICMR-National Institute for Research in Tuberculosis (Formerly Tuberculosis Research Centre), Chennai, Tamil Nadu, India; 11 ICMR-National Institute of Epidemiology, Chennai, Tamil Nadu, India; 12 Government Kilpauk Medical College, Chennai, Tamil Nadu, India; School of Digestive & Liver Diseases, Institute of Post Graduate Medical Education & Research, INDIA

## Abstract

**Background:**

We retrospectively data-mined the case records of Reverse Transcription Polymerase Chain Reaction (RT-PCR) confirmed COVID-19 patients hospitalized to a tertiary care centre to derive mortality predictors and formulate a risk score, for prioritizing admission.

**Methods and findings:**

Data on clinical manifestations, comorbidities, vital signs, and basic lab investigations collected as part of routine medical management at admission to a COVID-19 tertiary care centre in Chengalpattu, South India between May and November 2020 were retrospectively analysed to ascertain predictors of mortality in the univariate analysis using their relative difference in distribution among ‘survivors’ and ‘non-survivors’. The regression coefficients of those factors remaining significant in the multivariable logistic regression were utilised for risk score formulation and validated in 1000 bootstrap datasets.

Among 746 COVID-19 patients hospitalised [487 “survivors” and 259 “non-survivors” (deaths)], there was a slight male predilection [62.5%, (466/746)], with a higher mortality rate observed among 40–70 years age group [59.1%, (441/746)] and highest among diabetic patients with elevated urea levels [65.4% (68/104)]. The adjusted odds ratios of factors [OR (95% CI)] significant in the multivariable logistic regression were SaO_2_<95%; 2.96 (1.71–5.18), Urea ≥50 mg/dl: 4.51 (2.59–7.97), Neutrophil-lymphocytic ratio (NLR) >3; 3.01 (1.61–5.83), Age ≥50 years;2.52 (1.45–4.43), Pulse Rate ≥100/min: 2.02 (1.19–3.47) and coexisting Diabetes Mellitus; 1.73 (1.02–2.95) with hypertension and gender not retaining their significance. The individual risk scores for Sa**O**_**2**_<95–11, **U**rea ≥50 mg/dl-15, NL**R** >3–11, **A**ge ≥50 years-9, Pulse **R**ate ≥100/min-7 and coexisting **d**iabetes mellitus-6, acronymed collectively as ‘**OUR-ARDs score**’ showed that the sum of scores ≥ 25 predicted mortality with a sensitivity-90%, specificity-64% and AUC of 0.85.

**Conclusions:**

The ‘OUR ARDs’ risk score, derived from easily assessable factors predicting mortality, offered a tangible solution for prioritizing admission to COVID-19 tertiary care centre, that enhanced patient care but without unduly straining the health system.

## Introduction

The Coronavirus Disease 2019 (COVID-19) pandemic with its protean manifestation remains an unpredictable debacle; the spectrum of presentation varying from asymptomatic infection to a fulminant systemic inflammatory syndrome unleashed by the cytokine storm. Mortality has been 1.33%, in India, with 3,39,71,607 confirmed cases and 4,50,782 deaths, while Tamil Nadu, a southern state recorded 26,78,265 cases and 35,783 deaths, cumulative as of 11^th^ October 2021 [1].

Risk factors for mortality in COVID-19 reported in various studies included advanced age [2–13], male gender [6,7,9,11,13] and comorbidities [3,5,6,9–16] like diabetes mellitus, obesity, systemic hypertension, renal diseases, coronary artery disease [12] and malignancy. Apart from manifestations such as fever [8], cough [8], haemoptysis [2], dyspnoea [2,6,8], fatigue [8], loss of consciousness [2,6], laboratory parameters such as elevation of Neutrophil-to-Lymphocyte Ratio (NLR) [2,15], and increased levels of creatinine [3], lactate dehydrogenase (LDH) [2–4,10,12], direct bilirubin [2] and alanine aminotransferase [3], which provide early clues to the severity of disease, an increased plasma level of biomarkers like D-dimer [3–5,12], C-Reactive Protein (CRP) [6,7], serum ferritin [3], Interleukin-6 (IL-6) [3], and procalcitonin (PCT) fortifies these findings[3–5]. With the efficacy of specific antiviral and targeted immunomodulatory therapy still remaining elusive, prediction of mortality and risk stratification offers a rational approach for health resources allocation.

Hence, we retrospectively analysed data from a designated COVID-19 tertiary care centre in Tamil Nadu, South India, to unearth the risk factors predisposing to severe COVID-19 infection and mortality to formulate a risk score based on symptoms, comorbidities, vital signs and simple laboratory investigations, that will help to stratify patients into a high-risk group mandating preferential admission to tertiary care without undue delay, while safely retaining the low-risk group either at a primary care centre or at home, rendering the best care available, both clinically and psychosocially, ensuring the best survival advantage in both the groups, while simultaneously reducing the strain to the health care system.

## Methods

### Data collection

De-identified data from case records of COVID-19 confirmed patients (by real-time Reverse Transcription Polymerase Chain Reaction tests obtained from nasal or oropharyngeal swabs), hospitalized to a public sector COVID-19 tertiary care centre between 1^st^ May 2020 and 30^th^ November 2020 were analysed; the period roughly coinciding with the ascending slope of the epidemic curve in this region. Case records with insufficient data or without blood investigations were excluded. Institutional Ethics Committee approvals were duly obtained along with waiver from getting individual informed consents as the de-identified data were generated during the process of routine medical management, with the first recorded information on clinical status including comorbidities, peripheral oxygen saturation (SaO_2_), other vital parameters along with additional information on therapy and oxygen supplementation providing the raw data for analysis. Data were transcribed into an electronic format by two clinicians. An independent statistician checked the accuracy of data and analysed the data using IBM SPSS Statistics for Windows (IBM Corp. Released 2017, Version 25.0. Armonk, NY) and R software version 4.0.4 (R Core Team 2021).

### Categorisation of outcomes

Patients were broadly classified into ‘survivors’ (discharged or transferred to step-down COVID-19 care centres) and ‘non-survivors’ (deaths during hospitalisation) to derive prognostic factors determining mortality or recovery.

### Variables considered

The univariate analysis included demographic details describing age, gender, days to admission from the onset of symptoms and pre-existing comorbidities. Multiple comorbidities were present in some of the patients, which is not surprising by clinical parlance. Vital signs first recorded on admission that included SaO_2_, Pulse Rate, and Blood Pressure were copied along with basic laboratory reports consisting of plasma sugar (random), complete blood count, serum electrolytes, liver, and renal function tests. The relative difference in the symptomatology of COVID-19 between survivors and non-survivors was also explored to see if they played a role in envisaging future clinical courses.

Biomarkers like D-Dimer, Ferritin, PCT, and CRP levels were available only in a subset of the cohort. The receiver operating characteristic (ROC) curves of the above biomarkers were constructed and the area under the curves (AUC) was calculated and superimposed on the ROC curves of components used in the risk score that includes NLR, blood urea and SaO_2_ curves to provide and perceive their relative diagnostic accuracy with respect to these standard biomarkers. Since all patients were treated uniformly as per prevailing National guidelines [17], we probed only into the type of steroid used in treatment, and to determine if any specific steroid had an undue therapeutic advantage in combating COVID-19 triggered cytokine storm. Details of complications and treatment in the cohort along with the requirement of Oxygen supplementation are provided in the S1 Table.

### Statistical analysis and risk score construction

Univariate analysis of baseline variables was performed using the Chi-square test for proportions and Mann Whitney-U test for continuous variables, compared between survivors and non-survivors, to unearth candidate predictors among symptoms, comorbidities, vital signs, and basic lab investigations elucidated at admission, with the parameter being available in at least 20% of patients. The predictive model was built, applying the stepwise multivariable logistic regression, selecting those variables that were found significant at the 10% level in the univariate analysis. The ‘goodness of fit’ of the model was assessed using the Hosmer-Lemeshow test. The predictive capability was ascertained using the area under the curve of receiver operator characteristics (AUROC) and validated using the bootstrap method [18], fitted into 1000 bootstrap datasets. The risk score for each important parameter was arrived at, by multiplying the regression coefficient of the final model with ten and rounding it off to the nearest next integer; with the parameters remaining significant in at least 50% of the samples chosen from bootstrap data [19]. The capability of the risk score to predict mortality was once again endorsed by the AUROC value.

## Results

Of 746 case records of hospitalised patients, with near-complete details on clinical presentation including past history, comorbid conditions, symptoms at onset along with all baseline investigations available for prognosticating risk factors for mortality, there was a slight male preponderance in the occurrence of COVID-19 (62.5%, 466/746). There were 487 survivors and 259 non-survivors. The days to admission from the onset of symptoms were similar [median (IQR) 3(2–5) days] among survivors and non-survivors. The age group between 40 and 70 years was affected the most (59.1%, 441/746).

The distribution of symptoms among patients was as follows; fever—62.6% (455/727), breathlessness—49.9% (363/727), cough—46.4% (337/727), and gastrointestinal symptoms- 10.9% (79/727) in the decreasing order of frequency. Asymptomatic patients accounted for 6.7% (49/727).

There was at least one pre-existing comorbidity in 65.2% (475/728) of the cohort investigated with the major ones being diabetes mellitus (41.1%, 299/728), hypertension (30.1%, 219/728), cardiovascular disease (10.3%, 75/728), and kidney disease (9.9%, 72/728). Other comorbidities like malignancy, obesity and airway disease had a meagre representation in our cohort (<5%), precluding us from analysing their role further. Univariate analysis of baseline characteristics of the patients segregated into ‘survivors’ and ‘non-survivors’ is provided in Table 1.

**Table 1:** Baseline characteristics of hospitalised patients categorised into ‘survivors’ and ‘non-survivors’.

Variable		Study Population n = 746	Survivors n = 487	Non-Survivors n = 259	OR (95% CI)	P-value
**Demographics**						
Age (Years)^a^		50 (37–63)	44 (33–57)	62 (50–70)	1.07 (1.05, 1.08)	<0.01
Age (Years)	16–44	283 (37.9)	246 (50.5)	37 (14.3)	Reference	
	45–60	208(27.9)	139 (28.5)	69 (26.6)	3.26 (2.08, 5.12)	<0.001
	≥ 60	255(34.2)	102 (20.9)	153 (59.1)	9.85 (6.43, 15.1)	<0.001
Gender (Male)		466 (62.5)	293(60.2)	173 (66.8)	1.33 (0.97, 1.83)	0.075
Days to Admission^a^		3 (2–5)	3 (2–5)	3 (2–5)	0.96 (0.91, 1.02)	0.166
**Vital signs**						
Heart Rate (beats/min)^a^		92 (84–106)	90 (82–102)	98 (88–110)	1.03 (1.02, 1.04)	<0.001
SaO_2_ at Admission (%)^a^		96 (88–98)	98 (94–99)	88 (76.5–93.5)	0.88 (0.86, 0.9)	<0.001
SaO_2_ Levels	≥ 95%	407 (56.4)	352 (73.8)	55 (22. 5)	Reference	
	94–90%	114 (15.8)	60 (12.6)	54 (22.0)	5.76 (3.62, 9.17)	<0.001
	< 90%	201 (27.8)	65 (13.6)	136 (55.5)	13.91 (8.89, 20.18)	<0.001
**Symptomatology** ^**b1**^						
Symptoms		678 (93.3)	441 (91.3)	237 (97.1)	3.22 (1.43, 7.29)	0.003
Fever		455 (62.6)	303 (62.7)	152 (62.3)	0.98 (0.71, 1.35)	0.908
Headache		38 (5.2)	36 (7.5)	2 (0.8)	0.1 (0.02, 0.43)	<0.001
Cough		337 (46.4)	221 (45.8)	116 (47.5)	1.07 (0.79, 1.46)	0.649
Sore Throat		85 (11.7)	70 (14.5)	15 (6.2)	0.39 (0.22, 0.69)	0.001
Anosmia		42 (5.8)	39 (8.1)	3 (1.2)	0.14 (0.04, 0.46)	<0.001
Breathlessness		363 (49.9)	180 (37.3)	183 (75.0)	5.05 (3.58, 7.12)	<0.001
Chest Pain		12 (1.7)	4 (0.8)	8 (3.3)	4.06 (1.21, 13.62)	0.026
Haemoptysis		6 (0.8)	4 (0.8)	2 (0.8)	0.99 (0.18, 5.44)	0.999
Vomiting		26 (3.6)	17 (3.5)	9 (3.7)	1.05 (0.46, 2.39)	0.908
Myalgia		132 (18.2)	126 (26.1)	16 (6.6)	0.2 (0.12, 0.36)	<0.001
Abdominal Pain		79 (10.8)	53 (11.0)	26 (10.7)	0.97 (0.59, 1.59)	0.897
Altered Sensorium		27 (3.7)	8 (1.7)	19 (7.8)	5.01 (2.16, 11.63)	<0.001
**Co-morbid conditions** ^**b2**^						
Co-morbidities		475 (65.3)	261 (55.0)	214 (84.6)	4.5 (3.06, 6.62)	<0.001
Diabetes Mellitus		299 (41.1)	154 (32.4)	145 (57.3)	2.8 (2.04, 3.83)	<0.001
Hypertension		219 (30.1)	113 (23.8)	106 (42.0)	2.31 (1.67, 3.2)	<0.001
Cardiovascular Disease		75 (10.3)	33 (6.95)	42 (16.6)	2.67 (1.64, 4.33)	<0.001
Kidney Disease		72 (9.9)	22 (4.6)	50 (19.8)	5.07 (2.99, 8.6)	<0.001
Bronchial Asthma		25 (3.4)	21 (4.4)	4 (1.6)	0.35 (0.12, 1.02)	0.055
COPD		10 (1.4)	3 (0.6)	7 (2.8)	4.48 (1.15, 17.47)	0.038
Obesity		10 (1.4)	2 (0.4)	8 (3.2)	7.72 (1.63, 36.65)	0.004
Malignancy		10 (1.4)	2 (0.4)	8 (3.2)	7.72 (1.63, 36.65)	0.004
Liver Disease		8 (1.1)	1 (0.2)	7 (2.8)	13.49 (1.65, 110.25)	0.003
**Laboratory investigations [Median (IQR)]** ^**a**^						
Random Blood Sugar (mg/dL)		157 (97.5–280.0)	135 (93.0–244.5)	229.5 (124.5–342.5)	1 (1, 1.01)	<0.001
Urea (mg/dL)		32 (22.0–54.8)	27 (20.3–37.0)	56 (34–9)	1.03 (1.02, 1.04)	<0.001
Creatinine (mg/dL)		0.9 (0.8–1.3)	0.9 (0.8–1.1)	1.2 (0.9–1.8)	1.03 (0.99, 1.08)	<0.001
Haemoglobin (g/dL)		12.5 (10.9–13.7)	12.6 (11–13.8)	12.2 (10.6–13.6)	0.95 (0.88, 1.03)	0.144
Lymphocyte%		18.7 (10.7–31.0)	26 (15–35.5)	11 (5.5–18.0)	0.9 (0.88, 0.92)	<0.001
Neutrophil%		73.5 (60.8–83.4)	66.0 (56–78.4)	83.0 (75.7–9)	1.09 (1.07, 1.11)	<0.001
Absolute Lymphocyte count		1.5 (0.9, 2.2)	1.7 (1.2–2.5)	1.1 (0.7–1.6)	0.52 (0.38, 0.69)	<0.001
Absolute Neutrophil count		6.8 (4.4–10.5)	5.5 (3.7–8.7)	9.5 (6.7–13.5)	1.18 (1.11, 1.25)	<0.001
Neutrophil-to- Lymphocyte Ratio		3.9 (2.0, 7.8)	2.5 (1.6–5.2)	7.6 (4.1–16.1)	1.16 (1.12, 1.21)	<0.001
Platelet-Large Cell Ratio		26.5 (21.3–31.7)	26.0 (21.0–29.8)	27.4 (21.9–33.4)	1.17 (1.12, 1.23)	0.171

**Definition of Abbreviations:** COPD-Chronic Pulmonary Lung Disease, CI- Confidence Interval, p-Probability, IQR-Interquartile Range, SaO_2_-Peripheral Oxygen Saturation. Proportions are provided as percentages while other values are provided as Median (IQR). Ratios are provided as absolute numbers.

a—IQR (Interquartile Range).

b—Multiple symptoms and comorbidities may exist in the same patient and the sum may not add up to the total size of the cohort.

b1, n missing– 29

b2-n missing -28.

Symptoms such as anosmia [8.1% (39/483) vs 1.2% (3/244)], myalgia [24.2% (117/483) vs 6.1% (15/244)], and sore throat [14.5% (70/483) vs 6.1% (15/244)] favoured survival (p<0.001) while breathlessness [37.3% (180/483) vs 75% (183/244), p<0.001] and altered sensorium [1.7% (8/483) vs 7.8% (19/244), p<0.001] signalled a perilous outcome. There was not a single case of rhinorrhoea documented in our cohort.

Among hospitalised patients, survival was 84.6% (214/253) when there was no comorbidity, which reduced to 54.9% (261/475) when at least one comorbidity existed [p<0.001], which was further exacerbated by advancing age>50, [46.6% (149/320)]. There was a disproportionately higher incidence of mortality among diabetic patients compared to non-diabetic patients [48.5%, (145/299) vs 25.2%, (108/429)], p<0.001], the risk doubling with elevated urea levels compared to those with preserved renal function within the diabetic population [65.4%, (68/104) vs 27.3%, (33/121); p<0.001].

SaO_2_<90% at admission proved detrimental with a striking disparity perceived in the median (IQR) of SaO_2_ among survivors 98% (94–99%) compared to non-survivors 88% (76.5–93.5%). Tachycardia (PR ≥100/min) posed an ominous sign.

### Multivariable analysis and model creation

**Fig 1:**
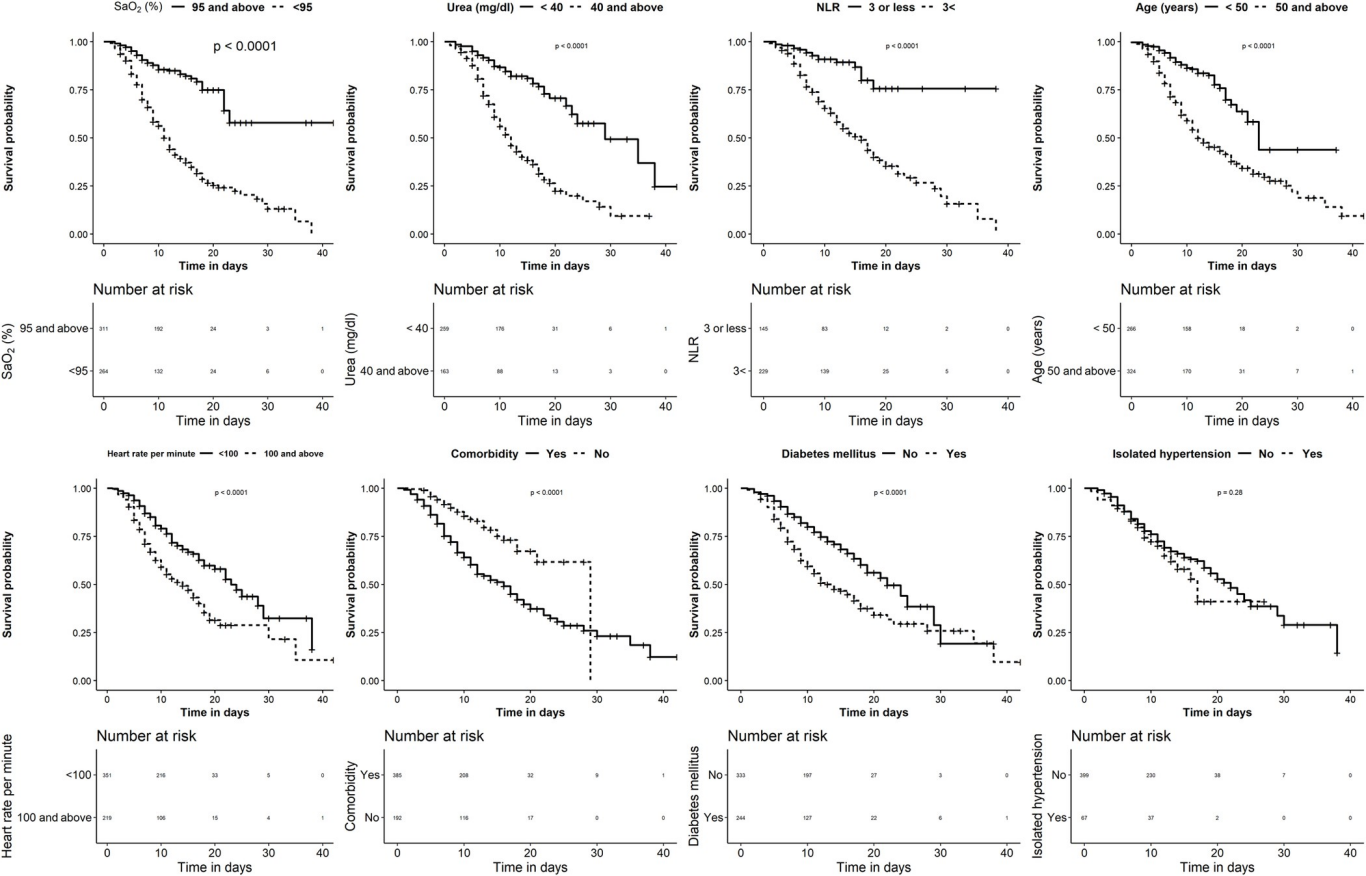
Kaplan-Meier analysis of the parameters significantly attributing to risk of mortality in the univariate analysis. Kaplan-Meier mean survival estimates for the time to mortality censored at 40 days. The numbers given above reflect the number of individuals who were alive at that particular time point in days, with or without the risk factors evaluated. Significance was computed using the log-rank test. **Definition of Abbreviations:** SaO_2_-Peripheral Oxygen Saturation, NLR-Neutrophil-to-Lymphocyte Ratio, BPM-Beats per minute.

The backward stepwise elimination logistic predictive model was fitted with clinically relevant candidate variables significant (p<0.1) in the univariate analysis and that could be feasible at a primary healthcare unit or screening centre. This included age, gender, SaO_2_, blood urea, pulse rate, diabetes, hypertension, and NLR. Fig 1 provides the influence of individual parameters on survival depicted as Kaplan-Meier survival curves. Gender and hypertension did not retain their significance in the multivariate logistic regression after adjustment for age and elevated urea and were subsequently excluded from the final model. The Hosmer-Lemeshow test (χ^2^ = 6.27, p = 0.617) established the goodness of fit of the model for this data. The AUC or the concordance index of 0.85 (95% CI: 0.81–0.89) indicated an adequate discriminating power, excluding overfitting bias in the model, which was further validated in the 1000 bootstrap datasets. The regression coefficients of parameters retained in the model were found to be significant in at least 70% of the bootstrap samples. The sign of the regression coefficient was also found to be consistent across the bootstrap samples. The multivariable logistic regression showing the adjusted odds ratio of the various parameters, with their regression coefficients used in deriving the risk score is provided in Table 2.

**Table 2:** Multivariable logistic regression showing the adjusted odds ratio of predictors influencing mortality, among COVID-19 infected patients admitted to hospital.

Variables	Multivariable Logistic Regression			Coefficients’ sign in bootstrap samples		Coefficients’ significance in bootstrap samples (%)	Numerical Risk Score
	B (SE)	p- value	Adjusted OR (95% CI)	+ (%)	- (%)		
Sa**O**_2_ (<95%)	1.09 (0.28)	<0.001	2.96 (1.71, 5.18)	100	0	97.5	11
**U**rea (≥50 mg/dL)	1.51 (0.29)	<0.001	4.51 (2.59, 7.97)	100	0	99.9	15
NL**R** (>3)	1.10 (0.33)	0.001	3.01 (1.61, 5.83)	100	0	94.6	11
**A**ge (≥50 years)	0.92 (0.28)	0.001	2.52 (1.45, 4.43)	100	0	92.6	9
Pulse **R**ate (≥100 BPM)	0.70 (0.27)	0.01	2.022 (1.19, 3.47)	100	0	83	7
**D**iabete**s** Mellitus (Yes)	0.55 (0.27)	0.044	1.73 (1.02, 2.95)	99.9	0.1	71.1	6

The risk score was constructed by multiplying each regression coefficient tagged to that parameter in the final model with ten and rounding it off to the nearest next integer with the parameters remaining significant in at least 50% of the samples chosen from bootstrap data.

**Definition of Abbreviations:** NLR-Neutrophil-to-Lymphocyte Ratio; SE-Standard Error, p- Probability; OR: Adjusted Odds Ratio, SaO_2_-Peripheral Oxygen Saturation. Variables significant in the univariate analysis were chosen of the multivariable logistic regression.

### Construction and calculation of risk score—‘OUR ARDs’ score

A regression coefficient-based scoring system, acronymed ‘**OUR-ARDs’** for easy recollection, was derived from the individual parameters that emerged significant for influencing mortality in the multivariate regression; the risk scores being: Sa**O**_2_ <95% - 11, **U**rea ≥50 mg/dl -15, NL**R**>3–11, **A**ge ≥50 years—9, Heart **R**ate ≥100 BPM—7 and history of **D**iabete**s** Mellitus—6. We elucidated that a sum of 25, as cut off, served as the critical value for predicting mortality with a sensitivity of 90% and specificity of 64%.

**Fig 2:**
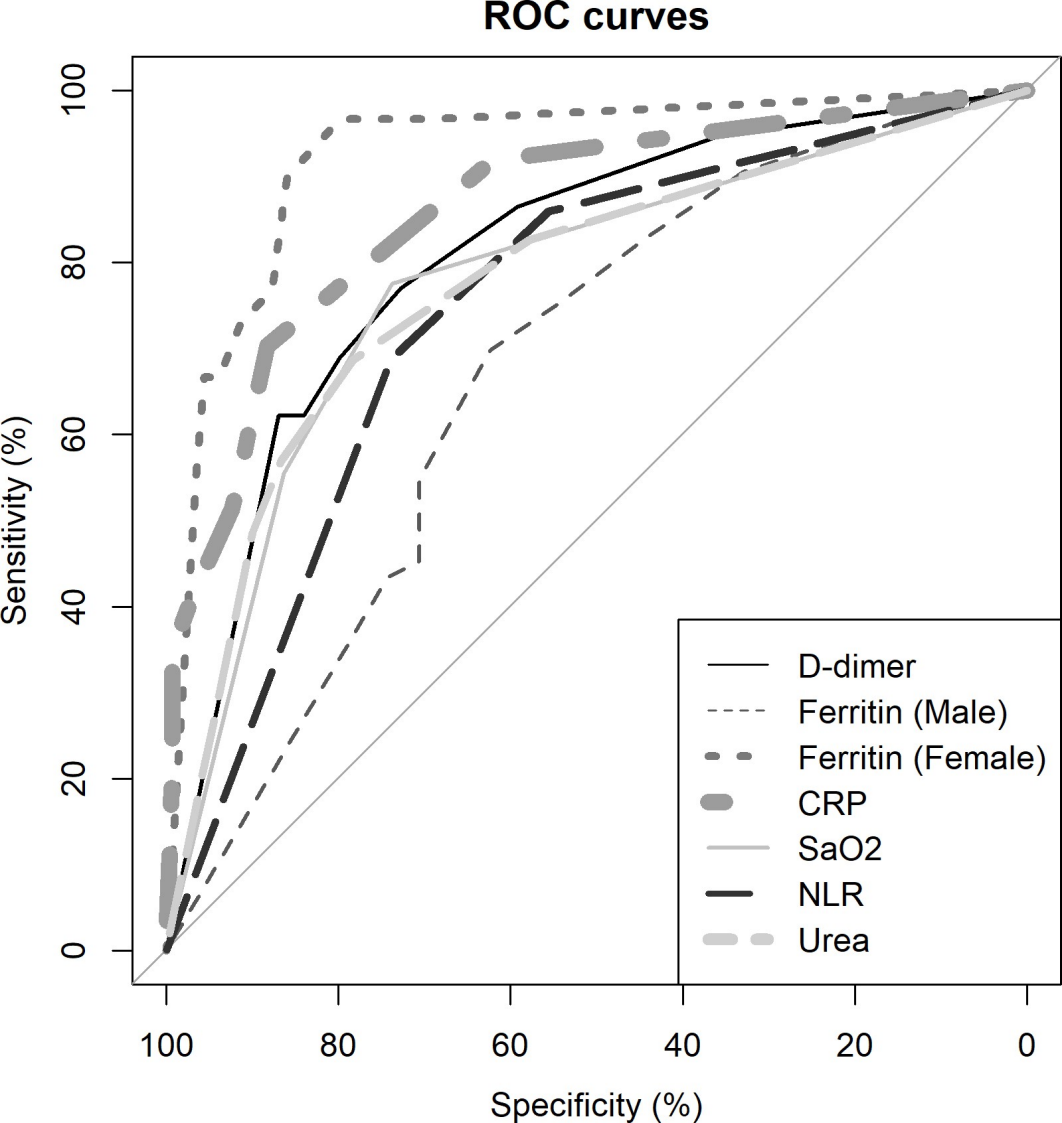
Illustrated depiction of ROC curves of important parameters used in score formulation along with biomarkers. The relative positions in the ROC curve also provide the precision of parameters, compared to biomarkers, in predicting mortality. **Definition of Abbreviations:** ROC Curve—Receiver Operating Characteristic Curve, CRP- C-Reactive Protein, SaO_2_—Peripheral Oxygen Saturation, NLR—Neutrophil-to-Lymphocyte Ratio.

Even though biomarkers were available only in a subset of patients, the AUROC values were robust that compelled us to provide an illustrated comparison of the parameters utilised for risk scores formulation in combination with the Biomarkers to understand their relative precision is provided in Fig 2.

On the therapeutic aspect of steroid selection, mortality rates were similar between usage of methylprednisolone (42.4%, 120/283) and dexamethasone (44.6%, 41/92), [p = 0.717].

## Discussion

The ‘OUR-ARDs’ score, an anamnesis of the underlying pathophysiology of COVID-19, showed that a score of ≥25 denoted a higher risk of mortality and needed priority admission to a tertiary care centre for escalation of management. Utilising clinically relevant determinants of mortality coupled with simple and basic laboratory investigations, gleaned from admission records of hospitalised COVID-19 patients, the OUR-ARDs score provides early clues and guidance in triaging of patients to the hospital, with the dual advantage of neither compromising on patient safety nor straining the health system. We found no difference between survivors and non-survivors with respect to time to admission from symptom onset, similar to other studies, suggesting a robust health system was in place [3,5].

While the SMART-COP and SCAP scores were operating at a higher or later level of medical care, oriented towards the transfer of in-patients in hospital wards to intensive care, our risk score was more focussed at the entry-level of health care, triaging patients into those who require a referral to a higher centre and those who can safely be managed at home or at the primary health care facility, which would prove useful when there is an exponential increase in admissions avoiding the congestion of the health system [20,21].

A comparative table of some of the risk scores aimed at triaging of RT-PCR positive COVID-19 patients, reported from various parts of the globe along with their translational application is briefly described for comparison and clinical utility as a ready reckoner in Table 3. While the risk scores such as the 4C mortality score [6] and the CALL score [10] were stratified with multiple levels, our risk score had a cut off level to decide on admission like the MuLBSTA score [22]. The clinical components of our score were similar to that of Galloway et al. [7] except for hypoalbuminemia as an additional factor in the latter.

**Table 3:** A concise comparison of risk scores used for triaging COVID-19 patients and their potential utilisation in clinical practice and public health.

Name of the Risk Score	Study Population	Components of Risk Score	Study Design, Region and Period of Study	AUC	Sensitivity	Specificity	Salient features of the score and utilisation
4C (Coronavirus Clinical Characterisation Consortium) Mortality Score Stephen R Knight et.al.	35463	Age, Sex, No. of Comorbidities, Respiratory Rate, SaO_2_, Consciousness (GCS) Urea, CRP>15, PPV of mortality - 0-3- low risk 4–8 Intermediate risk 9–14 high risk and above 15 –very high risk	Prospective, UK, Feb to Jun 2020.	-	99.7	98.8 (NPV)	Stratified risk score for 4 groups Easy to use score at hospital presentation for risk stratification and death in hospital,
CMR Tool Dimitris Bertsimas et.al	3927	Age 02<93% CRP>130 BUN> 18 Serum Creatinine >1.2	Retrospective study, USA, Greece, Italy, Spain	0.90	75	74	Predicting mortality among hospitalised patients, incorporating AI tools
Galloway JB et.al	1157	Older age Male sex Respiratory Rate Comorbidities Oxygenation Radiographic severity Higher neutrophils and CRP Lower albumin Renal impairment	Retrospective study, London, Mar-Apr 2020.				Mortality risk stratification, critical care admission and death–admission and discharge decisions
OUR-ARDs risk score Narendran G, Sumathi S et.al 2021	746	O-oxygen saturation -11 Urea>40 mg/dl-15 Ratio of NL>3–11 Age>50–9 Rate (Heart)>100–6 Diabetes-5 >25, Requires admission in tertiary care.	Retrospective analysis, India, May-Nov 2020.	0.85	90	64	Use of basic parameters that can be evaluated without sophisticated Lab investigations for Segregation and Prioritisation of patients for hospitalisation and retaining in a primary care facility
COVID-19 Scoring System (CSS) Yufeng Shang et.al.	452	Old-Age, CHD, LYMP%, PCT, D-Dimer	Retrospective from electronic medical records, China Jan-Mar 2020.	0.92	-	-	Prediction of inpatient mortality and complications
CALL Score Dong Ji et.al.	208	Comorbidity for at least 6 months, Age, Lymphocyte, LDH (4 to 13 points Up to 6 –no progression	Retrospective study, China, Jan-Feb 2020	0.91	95	78	Stratified risk score, 3 levels, with just 4 factors that could be easily evaluated and managed safely at district hospitals for disease progression or referral to tertiary care
SOFA / qSOFA Score (quick Sequential Organ Failure Assessment Score) Sijia Liu et.al.	127	systolic blood pressure ≤ 100 mmHg, Respiratory rate ≥ 22 breaths/min, altered mental status.	Retrospective study, China, Jan-Mar 2020	0.89 (SOFA) 0.74 (qSOFA)	90	83.1	Critically ill patients–escalate therapy, referral to ICU and hospital mortality, more useful in advanced age
MuLBSTA Score Mukul Preetam et.al.	122	Multi-lobular involvement, Absolute lymphocyte count, Bacterial co-infection, Smoking history, H/o Hypertension, Age >12 risk score associated with increased mortality.	Retrospective study, India, July-Aug 2020.	122	-	-	14-day mortality risk score in Indian population and need for ICU admission
NEWS2 (National Early Warning Score 2) Myrstad M et.al.	66	Respiratory Rate, SaO_2_, Systolic BP, Pulse Rate, Body Temperature, Level of Consciousness Need for supplemental oxygen > 6 –clinical deterioration Age combined as adapted NEWS2 score	Prospective cohort study, Norway, Mar-Apr 2020.	0.82	80%	84.3%	Scoring for clinical deterioration in acutely ill patients. Both hypoxemia and need for supplemental oxygenation taken into consideration to capture “silent hypoxia”.

The risk scores are not all inclusive. Those that were aimed at triaging among RT-PCR positive COVID-19 patients, reported from various parts of the globe along with their translation application is briefly described as a ready reckoner for comparison. **Definition of Abbreviations:** NPV—Negative Predictive Value, GCS—Glasgow Coma Scale, CHD—Coronary Heart Disease, ICU- Intensive Care Unit, BP- Blood Pressure.

Exploring the individual components of the risk score, levels of SaO_2_ at admission critically determined survival [23] apart from elevated urea and NLR. Tachycardia, (heart rate >100/min) had been a well-known prognostic factor in pneumonia, with COVID-19 being no exception. However, the cut-offs for heart rate varied among individual studies [24].

An intact functioning kidney is often a prerequisite for survival in COVID-19. Elevated Urea above 40 mg/dl was a strong predictor of mortality, frequented more commonly in the background of chronic kidney disease proving detrimental to survival. Renal impairment has been associated with delayed clearance of cytokines, along with elevated levels of von Willebrand factor (vWF), adding to both exaggerated inflammatory response and pro-coagulable state. Pre-existing vasodynamic insults caused by underlying diabetes mellitus or hypertension further exacerbated the condition [25,26]. Mudatsir et al. proclaimed that elevated levels of urea levels confronted at the time of COVID-19 were due to the direct involvement of renal cells by the Coronavirus rather than due to pre-existing renal disease which puts any patient with an increasing urea level into the high-risk category irrespective of the background renal status [27].

An elevated NLR, as in other viral infections, carried a poor prognosis, serving as a surrogate indicator for survival and the need for ventilator support [15,28]. In COVID-19, the increase in neutrophil number, after infiltration, generated greater quantities of cytokines, leading to immune fury and multi-systemic inflammation, ending fatally. The study by Zhou C et al. confirmed that profound lymphopenia was the strongest influencer of mortality and rightfully retained a higher score for severity in our study equivalent to blood oxygen saturation. (Table 2). Even among healthy young individuals without any comorbidities, a higher NLR and lymphopenia <900 cells/mm^3^ predicted a poor prognosis [29]. The reasons postulated for this corresponding decrease in lymphocyte number include a reduction of circulating lymphocytes, apoptotic destruction, decreased production in the spleen and thymus due to direct involvement by the virus, and the negative feedback signals received from elevated plasma levels of IL-6. The meta-analysis by Huang W et al. showed a profound decline in the entire repertoire of the lymphocyte family in COVID-19 including CD4+, CD8+, B-cells and NK-cells [30].

Age ≥50 years was an independent factor contributing to severity. Survivors were two decades younger than Non-Survivors in our study and this feature has been commonly reported across all studies (Table 3). Various studies established a linear relationship between the severity of COVID-19 and the increasing number of comorbidities in the same individual, supplemented by advancing age [6,31]. Our study elucidated that the diabetic patients with concomitant elevated renal parameters were the most likely to succumb to COVID-19. Hypertension and diabetes exacerbate many viral infections, with higher mortalities reported in dengue, Yellow Nile Fever and Zika Virus infection [32]. Our data did show that both isolated systemic hypertension and cardiovascular disease to be of higher significance in the univariate analysis (Table 1). However, hypertension did not retain its significance in the multivariable analysis, after adjusting for age and elevated urea. This aspect needs careful interpretation in lieu of possible overlap and interdependence between hypertension and other factors like age, diabetes, and the tendency for renal compromise or insufficiency, possibly mitigating the attributable role of hypertension individually in causing COVID-19 related mortality in our study.

The mortality rate was less than one per cent in the absence of any comorbidity while patients with solid organ transplants formed the other end of the spectrum having the greatest causality [33]. Underlying malignancies, associated cardiovascular diseases [34], diabetes, COPD, and hypertension have been reported as important determinants of survival in various other studies [31]. Incidentally, some of these did not have sufficient representation in our study population to draw a firm conclusion.

Venturing into the symptomatology of COVID-19, breathlessness and altered sensorium at admission were logically linked to a poorer prognosis in line with the findings of Myrstad et al. [35], while anosmia, myalgia, and sore throat offered a better chance of survival. Certain symptoms like fever, cough, abdominal pain and vomiting were non-committal in predicting survival similar to the report by Dependra et al. [15]. Intriguingly, rhinorrhoea among COVID19 patients was a rarity [29]. This categorisation of COVID-19 symptomatology however could be ‘wave-specific’ and needs to be interpreted in the light of region-specific association with other objective findings such as SaO_2_, NLR, etc. for better clinical application. A meta-analysis from Indonesia found that patients with breathlessness, anorexia, and fatigue had a worse prognosis [27].

Fig 2 depicts the AUROC of the components of the OUR-ARDs risk score superimposed with biomarkers estimated in a subset of our population, to bring out the comparative precision of the components of the risk score with the former. Based on clinical intuition, we hypothesize that those reaching risk scores above 19 but below 25 may be re-evaluated periodically using the same risk score for maximising patient safety or additionally correlated with biomarker estimation to precisely estimate the risk (S2 Table) as biomarkers strengthen the decision.

Therapeutically, we found no differences between the efficacies of intravenous steroids namely dexamethasone and methylprednisolone in preventing mortality. However, other studies have shown the latter to be associated with a lesser requirement of ventilator support and a shorter stay in hospital [36,37].

The strength of this study lies in the utilisation of basic parameters, easily available and readily assessable even in a peripheral hospital without the requirement of complex medical gadgets/tests for predicting mortality and formulation of a risk score from the statistically significant factors. We had avoided using symptoms in the multivariate analysis due to its subjective nature, inherent heterogeneity, with those symptoms crucially determining mortality presenting late during the course of the disease along with contradictory features like apparent “silent hypoxia”, taking the physician by surprise. The study has important limitations. Selection bias and missing data were inevitable with the pandemic still on a rampage. The internal validation was done using the bootstrap method that had provided reliable estimates for the predictive model. With the analysis dictated by the availability of data, we could confidently allude those who were not included in the analysis (data unavailable in hospital records) predominantly belonged to asymptomatic, incidental, or milder cases of COVID-19, who did not require much medical attention as such. Data on biomarkers were limited as priority was given to the higher risk group at the time when patients were exponentially pouring in. But the AUC values of the biomarkers were robust that we could illustrate the accuracy of the components of risk scores concerning crucial biomarkers. A recent review by Rod JE et al. showed only CRP and D-dimer to be the consistently performing predictors across studies [38]. Considering these limitations and that the risk score had to be applied at the entry into the health care system, where a physician should take a crucial decision whether to refer to tertiary care or retain the patients at a primary health care system, where biomarkers may not be readily available, we had not included them in the current model suggested. However, we do admit that the biomarkers D-Dimer and CRP could greatly enhance the accuracy of the risk score. Even though other comorbidities could potentially have a bearing on COVID-19 survival, it was difficult to ascertain their influence on COVID-19 severity due to the smaller numbers available in the current cohort study. For instance, a study reported that asthmatics had a better outcome compared to COPD patients [4]. Our data does not truly represent case fatality or true mortality rates, as data was extracted from a portion of patients with near-complete clinical history and baseline investigations. With the available data, our risk score may not be directly translatable to post-COVID-19 extra-pulmonary complications that may also demand admission. Regional, strain and ethnic variations in COVID-19 presentation may have to be accounted for, while interpreting the results.

## Conclusion

With the compelling global travel and COVID-19 sparing no country, predictors of COVID-19 fatality and stratification of risk using a simplified risk score could provide a tangible solution to rationalise resource allocation for best results and efficient patient recovery, importantly without undue strain on the health system.

## Supporting information

S1 Table Details of complications and treatment in the cohort along with supplementation of O_2_. ^a^-Heparin includes both Low Molecular Weight Heparin (LMWH) and unfractionated heparin (UFH).
(DOCX)

S2 Table Summated OUR-ARDs risk scores and their corresponding sensitivity and specificity. The OUR-ARDs risk score calculated using the 6 parameters namely, Peripheral Oxygen Saturation, Urea, Neutrophil-to-Lymphocyte Ratio, Age, Heart Rate and Diabetes Mellitus are provided, along with the sensitivity and specificity of each of the risk scores.
(DOCX)

S1 STROBE statement The STROBE checklist of items for this manuscript. (PDF)

S1 File (XLSX)

## Abbreviations

AUC: Area Under the Curve
AUROC: Area Under the Receiver Operating Characteristic
CI: Confidence Interval
COPD: Chronic Obstructive Pulmonary Disease
COVID-19: Coronavirus Disease-2019
CRP: C-reactive protein
IL: Interleukin
IQR: Interquartile Range
NLR: Neutrophil-to-Lymphocyte Ratio
OR: Adjusted Odds Ratio
p: Probability
PCT: Procalcitonin
ROC: Receiver Operating Characteristic
RT-PCR: Reverse Transcription-Polymerase Chain Reaction
SaO2: Peripheral Oxygen Saturation
SE: Standard Error
vWF: von Willebrand Factor
