# Supplementary material for: Predictors of mortality among hospitalized COVID-19 patients and risk score formulation for prioritizing tertiary care—An experience from South India
Source: PLoS One. 2022 Feb 3;17(2):e0263471. doi: 10.1371/journal.pone.0263471 (PMC8812932; doi:10.1371/journal.pone.0263471)
Supplement: S1 Table — a-Heparin includes both Low Molecular Weight Heparin (LMWH) and unfractionated heparin (UFH). (DOCX) [file pone.0263471.s001.docx]

**S1 Table. Details of complications and treatment in the cohort along with supplementation of O_2_**

| **Variable** | | **Survivors**  **N (%)** | **Non-Survivors**  **N (%)** | **P-value** |
| --- | --- | --- | --- | --- |
| Acute Lung Injury /  Acute Respiratory Distress Syndrome | | 0 (0.0) | 44 (17.0) | <0.001 |
| Acute Kidney Injury | | 1 (0.2) | 17 (2.3) | <0.001 |
| Sepsis / Multi-Organ Dysfunction | | 0 (0.0) | 26 (10.0) | <0.001 |
| Antibiotics | | 420 (86.2) | 232 (93.9) | 0.002 |
| Specific Antivirals | | 162 (34.0) | 95 (42.0) | 0.038 |
| Steroids | | 237 (48.7) | 193 (79.2) | <0.001 |
| Heparin^a^ | | 223 (45.9) | 182 (74.3) | <0.001 |
| Drug - Ivermectin | | 14 (2.9) | 7 (2.8) | 0.975 |
| Drug - Hydroxychloroquine | | 47 (9.7) | 14 (5.7) | 0.065 |
| O_2_ Supplementation | | 141 (29.1) | 220 (92.4) | <0.001 |
| Mode of O_2_ Delivery | Simple Face Mask | 67 (13.8) | 87 (36.6) |  |
|  | Non-Rebreather Mask | 58 (12.0) | 33 (13.9) |  |
|  | High Flow Nasal Cannula | 15 (3.1) | 70 (29.4) |  |
|  | Mechanical Ventilation | 1 (0.) | 30 (12.) |  |

^a^Heparin includes both Low Molecular Weight Heparin (LMWH) and unfractionated heparin (UFH).
