# Supplementary material for: Predictors of mortality among hospitalized COVID-19 patients and risk score formulation for prioritizing tertiary care—An experience from South India
Source: PLoS One. 2022 Feb 3;17(2):e0263471. doi: 10.1371/journal.pone.0263471 (PMC8812932; doi:10.1371/journal.pone.0263471)
Supplement: S2 Table — The OUR-ARDs risk score calculated using the 6 parameters namely, Peripheral Oxygen Saturation, Urea, Neutrophil-to-Lymphocyte Ratio, Age, Heart Rate and Diabetes Mellitus are provided, along with the sensitivity and specificity of each of the risk scores. (DOCX) [file pone.0263471.s002.docx]

**S2 Table. Summated OUR-ARDs risk scores and their corresponding sensitivity and specificity**

| **Risk score summated** | **Sensitivity** | **Specificity** | **Risk score summated** | **Sensitivity** | **Specificity** |
| --- | --- | --- | --- | --- | --- |
| 3 | 0.99 | 0.16 | 30 | 0.77 | 0.73 |
| 7 | 0.99 | 0.19 | 31 | 0.77 | 0.74 |
| 8 | 0.99 | 0.27 | 32 | 0.74 | 0.78 |
| 10 | 0.99 | 0.3 | 34 | 0.70 | 0.81 |
| 12 | 0.99 | 0.38 | 36 | 0.65 | 0.84 |
| 14 | 0.98 | 0.39 | 38 | 0.54 | 0.9 |
| 16 | 0.97 | 0.44 | 39 | 0.51 | 0.92 |
| 17 | 0.97 | 0.45 | 40 | 0.5 | 0.92 |
| 18 | 0.96 | 0.46 | 42 | 0.48 | 0.93 |
| 19 | 0.95 | 0.53 | 43 | 0.47 | 0.93 |
| 21 | 0.93 | 0.56 | 44 | 0.46 | 0.94 |
| 22 | 0.93 | 0.57 | 45 | 0.35 | 0.95 |
| 23 | 0.92 | 0.62 | 47 | 0.29 | 0.97 |
| 25 | 0.90 | 0.64 | 49 | 0.29 | 0.97 |
| 27 | 0.81 | 0.69 | 51 | 0.27 | 0.97 |
| 28 | 0.79 | 0.7 | 53 | 0.16 | 0.98 |
| 29 | 0.78 | 0.72 | 56 | 0.09 | 0.99 |

The OUR-ARDs risk score calculated using the 6 parameters namely, Peripheral Oxygen Saturation, Urea, Neutrophil-to-Lymphocyte Ratio, Age, Heart Rate and Diabetes Mellitus are provided, along with the sensitivity and specificity of each of the risk scores.
