# Supplementary material for: Predictors of mortality among hospitalized COVID-19 patients and risk score formulation for prioritizing tertiary care—An experience from South India
Source: PLoS One. 2022 Feb 3;17(2):e0263471. doi: 10.1371/journal.pone.0263471 (PMC8812932; doi:10.1371/journal.pone.0263471)
Supplement: S1 STROBE statement — (PDF) [file pone.0263471.s003.pdf]

S3 STROBE Statement - checklist of items that should be included in reports of observational studies.

Checklist	Item No	Recommendation	Remarks
**Title and abstract**	1	(*a*) Indicate the study's design with a commonly used term in the title or the abstract	Title and abstract
		(*b*) Provide in the abstract an informative and balanced summary of what was done and what was found	Author Summary and Abstract - Methods & Findings
**Introduction**			
Background/rationale	2	Explain the scientific background and rationale for the investigation being reported	Introduction - paragraphs 1-3
Objectives	3	State specific objectives, including any prespecified hypotheses	Introduction - paragraph 3
**Methods**			
Study design	4	Present key elements of study design early in the paper	Methods - Data collection & categorisation of outcomes subsections
Setting	5	Describe the setting, locations, and relevant dates, including periods of recruitment, exposure, follow-up, and data collection	Methods - Data collection subsection
Participants	6	(*a*) *Cohort study*—Give the eligibility criteria, and the sources and methods of selection of participants. Describe methods of follow-up<br>*Case-control study*—Give the eligibility criteria, and the sources and methods of case ascertainment and control selection. Give the rationale for the choice of cases and controls<br>*Cross-sectional study*—Give the eligibility criteria, and the sources and methods of selection of participants	Methods - Data collection subsection
		(*b*) *Cohort study*—For matched studies, give matching criteria and number of exposed and unexposed<br>*Case-control study*—For matched studies, give matching criteria and the number of controls per case	Section not applicable
Variables	7	Clearly define all outcomes, exposures, predictors, potential confounders, and effect modifiers. Give diagnostic criteria, if applicable	Methods - Variables considered subsection and Table 1
Data sources/ measurement	8*	For each variable of interest, give sources of data and details of methods of assessment (measurement). Describe comparability of assessment methods if there is more than one group	Methods - statistical analysis and risk score construction subsection (only one group considered)
Bias	9	Describe any efforts to address potential sources of bias	Results - Multivariable analysis and model creation<br>Discussion - paragraph 12 as

			limitations
Study size	10	Explain how the study size was arrived at	Not applicable as the study was only a retrospective analysis
Quantitative variables	11	Explain how quantitative variables were handled in the analyses. If applicable, describe which groupings were chosen and why	Methods – Categorisation of Outcomes and variables considered subsections
Statistical methods	12	(*a*) Describe all statistical methods, including those used to control for confounding	Methods – Statistical analyses And risk score construction
		(*b*) Describe any methods used to examine subgroups and interactions	Methods – Statistical analyses And risk score construction
		(*c*) Explain how missing data were addressed	Addressed as a limitation
		(*d*) *Cohort study*—If applicable, explain how loss to follow-up was addressed<br>*Case-control study*—If applicable, explain how matching of cases and controls was addressed<br>*Cross-sectional study*—If applicable, describe analytical methods taking account of sampling strategy	Methods – Statistical analyses And risk score construction
		(*e*) Describe any sensitivity analyses	Section not applicable
**Results**			
Participants	13*	(a) Report numbers of individuals at each stage of study—eg numbers potentially eligible, examined for eligibility, confirmed eligible, included in the study, completing follow-up, and analysed	Results (paragraph 1) and Table 1
		(b) Give reasons for non-participation at each stage	Section not applicable
		(c) Consider use of a flow diagram	Section not applicable
Descriptive data	14*	(a) Give characteristics of study participants (eg demographic, clinical, social) and information on exposures and potential confounders	Table 1 and Supplementary Table 1
		(b) Indicate number of participants with missing data for each variable of interest	Section not applicable
		(c) *Cohort study*—Summarise follow-up time (eg, average and total amount)	Section not applicable
Outcome data	15*	*Cohort study*—Report numbers of outcome events or summary measures over time<br>*Case-control study*—Report numbers in each exposure category, or summary measures of exposure<br>*Cross-sectional study*—Report numbers of outcome events or summary measures	Section not applicable
Main results	16	(*a*) Give unadjusted estimates and, if applicable, confounder-adjusted estimates and their precision (eg, 95% confidence interval). Make clear which confounders were adjusted for and why they were included	Table 1 and Results section (paragraphs 1-6)
		(*b*) Report category boundaries when continuous	Results section (paragraphs 1-6)

		variables were categorized	
		(*c*) If relevant, consider translating estimates of relative risk into absolute risk for a meaningful time period	Figure 1 and Table 2
Other analyses	17	Report other analyses done—eg analyses of subgroups and interactions, and sensitivity analyses	Results – Multivariable analysis & model creation and construction & calculation of risk score subsections, Figure 2 and Tables 2 & 3
**Discussion**			
Key results	18	Summarise key results with reference to study objectives	Discussion (paragraphs 1 - 11)
Limitations	19	Discuss limitations of the study, taking into account sources of potential bias or imprecision. Discuss both direction and magnitude of any potential bias	Discussion (paragraph 7)
Interpretation	20	Give a cautious overall interpretation of results considering objectives, limitations, multiplicity of analyses, results from similar studies, and other relevant evidence	Discussion (paragraph 12)
Generalisability	21	Discuss the generalisability (external validity) of the study results	Discussion (paragraph 12)
**Other information**			
Funding	22	Give the source of funding and the role of the funders for the present study and, if applicable, for the original study on which the present article is based	No funding available

*Give information separately for cases and controls in case-control studies and, if applicable, for exposed and unexposed groups in cohort and cross-sectional studies.

Note: An Explanation and Elaboration article discusses each checklist item and gives methodological background and published examples of transparent reporting. The STROBE checklist is best used in conjunction with this article (freely available on the Web sites of PLoS Medicine at http://www.plosmedicine.org/, Annals of Internal Medicine at http://www.annals.org/, and Epidemiology at http://www.epidem.com/). Information on the STROBE Initiative is available at www.strobe-statement.org.
